# Radiomics signatures for predicting the Ki-67 level and HER-2 status based on bone metastasis from primary breast cancer

**DOI:** 10.3389/fcell.2023.1220320

**Published:** 2024-01-08

**Authors:** Hongxiao Zhang, Shuxian Niu, Huanhuan Chen, Lihua Wang, Xiaoyu Wang, Yujiao Wu, Jiaxin Shi, Zhuoning Li, Yanjun Hu, Zhiguang Yang, Xiran Jiang

**Affiliations:** ^1^ School of Intelligent Medicine, China Medical University, Shenyang, Liaoning, China; ^2^ Department of Oncology, Shengjing Hospital of China Medical University, Shenyang, Liaoning, China; ^3^ Department of Radiology, Cancer Hospital of China Medical University, Liaoning Cancer Hospital and Institute, Shenyang, Liaoning, China; ^4^ Department of Medical Imaging, Cancer Hospital of China Medical University, Liaoning Cancer Hospital and Institute, Shenyang, Liaoning, China; ^5^ Department of Radiology, Shengjing Hospital of China Medical University, Shenyang, Liaoning, China

**Keywords:** breast cancer, spinal metastasis, HER-2, Ki-67, radiomics

## Abstract

This study explores the potential of radiomics to predict the proliferation marker protein Ki-67 levels and human epidermal growth factor receptor 2 (HER-2) status based on MRI images of patients with spinal metastasis from primary breast cancer. A total of 110 patients with pathologically confirmed spinal metastases from primary breast cancer were enrolled between Dec. 2017 and Dec. 2021. All patients underwent T1-weighted contrast-enhanced MRI scans. The PyRadiomics package was used to extract features from the MRI images based on the intraclass correlation coefficient and least absolute shrinkage and selection operator. The most predictive features were used to develop the radiomics signature. The Chi-Square test, Fisher’s exact test, Student’s *t*-test, and Mann–Whitney U test were used to evaluate the clinical and pathological characteristics between the high- and low-level Ki-67 groups and the HER-2 positive/negative groups. The radiomics models were compared using receiver operating characteristic curve analysis. The area under the receiver operating characteristic curve (AUC), sensitivity (SEN), and specificity (SPE) were generated as comparison metrics. From the spinal MRI scans, five and two features were identified as the most predictive for the Ki-67 level and HER-2 status, respectively. The developed radiomics signatures generated good prediction performance for the Ki-67 level in the training (AUC = 0.812, 95% CI: 0.710–0.914, SEN = 0.667, SPE = 0.846) and validation (AUC = 0.799, 95% CI: 0.652–0.947, SEN = 0.722, SPE = 0.833) cohorts. Good prediction performance for the HER-2 status was also achieved in the training (AUC = 0.796, 95% CI: 0.686–0.906, SEN = 0.720, SPE = 0.776) and validation (AUC = 0.705, 95% CI: 0.506–0.904, SEN = 0.733, SPE = 0.762) cohorts. The results of this study provide a better understanding of the potential clinical implications of spinal MRI-based radiomics on the prediction of Ki-67 levels and HER-2 status in breast cancer.

## 1 Introduction

Breast cancer (BC) is the most common form of cancer worldwide, and has exhibited an increasing incidence trend in recent years (Ye et al., 2020; Loibl et al., 2021). Early and appropriate treatment are warranted to increase the 5-year survival rates of BC patients (Allemani et al., 2015). The status of the molecular hallmarks of BC are critical for prognosis and treatment, and have been extensively characterized (Cheang et al., 2009). The human epidermal growth factor receptor 2 (HER-2) status and proliferation marker protein Ki-67 levels are two crucial factors in determining the treatment strategy for BC patients (Yerushalmi et al., 2010; Loibl and Gianni, 2017). HER-2 displays amplification or protein overexpression in 20%–30% of BC cases, and is important for the determination of therapy strategies (Loibl and Gianni, 2017). BC patients that are HER-2 positive usually have a high likelihood of achieving a pathological complete response (pCR) after the neoadjuvant treatment and generating favorable outcomes (van Ramshorst et al., 2017). Ki-67 is an independent prognostic characteristic reflecting the extent of proliferative activity (Yerushalmi et al., 2010). High Ki-67 expression levels are associated with more aggressive tumor growth and poorer prognosis (Wiesner et al., 2009; Yerushalmi et al., 2010). Patients that are HER-2 positive (van Ramshorst et al., 2017) and/or have a low Ki-67 expression level (<14%) (Kim et al., 2014) are usually advised to undergo adjuvant chemotherapy. Therefore, early and accurate evaluation of the Ki-67 level and HER-2 status is essential for individual therapy decisions.

Many BC patients suffer from metastasis, with bone as the most frequent metastatic site (Hagberg et al., 2013; Foerster et al., 2015). Spinal metastasis is a major cause of severe morbidity for BC (Janjan et al., 2009). When the primary BC is unavailable, spinal metastasis provides an important alternative for identifying the tumor characteristics of the primary BC (Weigelt et al., 2005). However, clinical routine assessment of the Ki-67 expression and HER-2 status is based on immunohistochemistry (IHC) (Gnant et al., 2011), which relies on a punch biopsy. This is an invasive diagnostic procedure that is dangerous to perform on the spinal column because of the potential to damage the nerves (Loibl et al., 2021). Although magnetic resonance imaging (MRI) is commonly used as a noninvasive imaging method for confirming the existence of spinal metastases, there is still no specific marker that can be recognized by visual inspection of MRI images as reflecting the Ki-67 level or HER-2 status.

Recently, radiomics has emerged as a method that may enable the profiling of tumor characteristics in a noninvasive manner by extracting and analyzing large numbers of quantitative features (Gillies et al., 2016). Radiomics-based computer-aided diagnosis allows for the quantitative extraction and selection of valuable features from medical imaging, providing a powerful noninvasive tool in oncology research (Lambin et al., 2017; Hosny et al., 2018). Many studies have analyzed the correlations between MRI-based radiomics and molecular subtypes in BC (Sutton et al., 2016; Fan et al., 2017; Fan et al., 2019; Leithner et al., 2020; Li et al., 2021; Niu et al., 2022). Previous studies have proposed the radiological differentiation of molecular subtypes based on the primary BC. To the best of our knowledge, radiological characterization for the identification of Ki-67 and HER-2 status based on bone metastasis has not been evaluated. Therefore, the purpose of this study is to investigate the potential of MRI-based radiomics for predicting the Ki-67 level and HER-2 status on spinal bone metastasis from primary BC.

## 2 Methods

### 2.1 Patients

**FIGURE 1:**
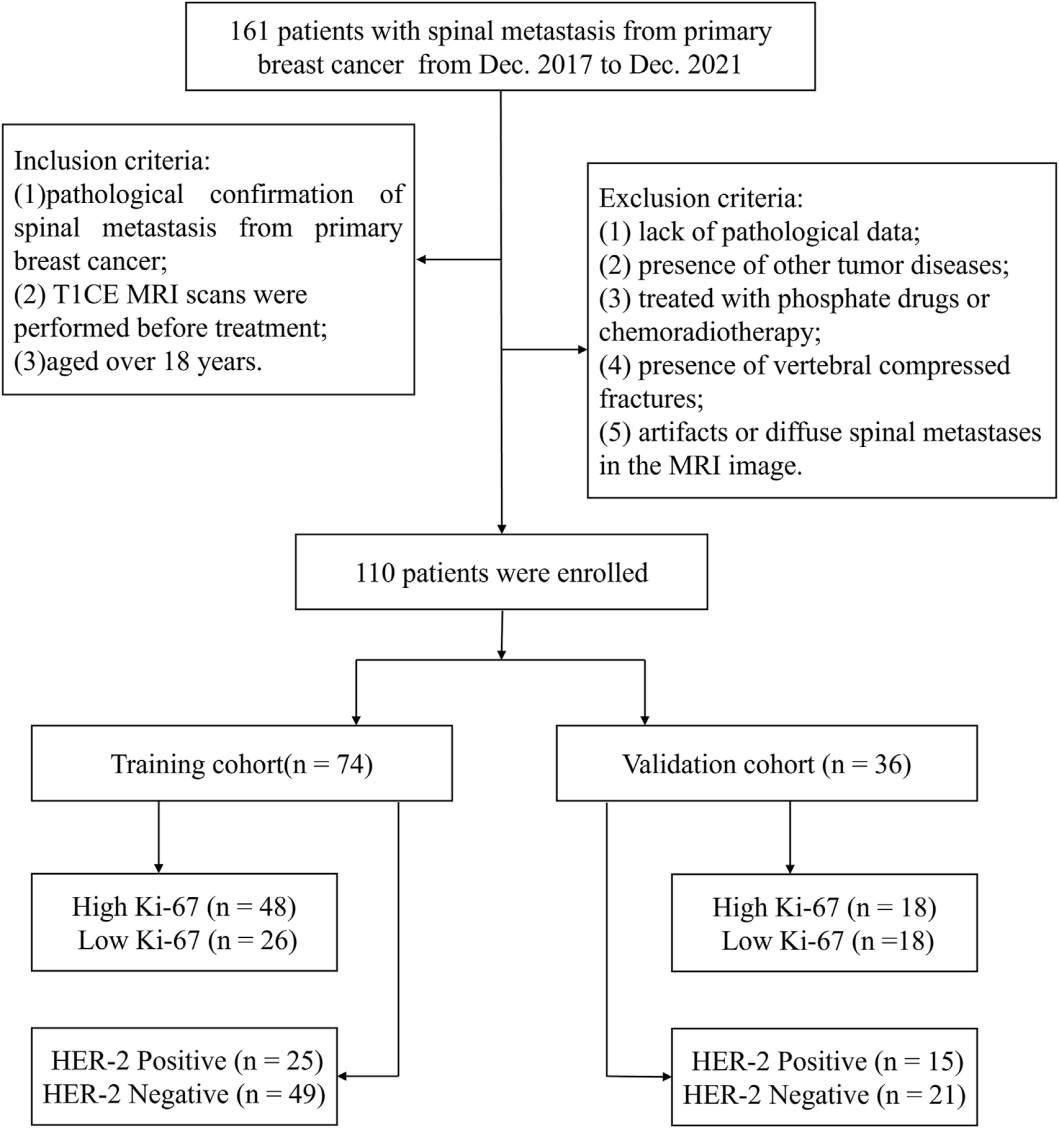
Patient recruitment in this study.

Retrospective research was approved by the ethics committee of our hospital, with the informed consent requirement waived because of the retrospective nature. This study was conducted between Dec. 2017 and Dec. 2021, and included data from 110 patients diagnosed with spinal metastasis from primary BC. The patients were enrolled according to the following inclusion criteria: 1) pathological confirmation of spinal metastasis from primary BC, 2) T1-weighted contrast-enhanced (T1CE) MRI scans were performed before treatment, and 3) aged over 18 years. The exclusion criteria were: 1) lack of pathological data, 2) presence of other tumor diseases, 3) treated with phosphate drugs or chemoradiotherapy, 4) presence of vertebral compressed fractures, and 5) artifacts or diffuse spinal metastases in the MRI image. The included patients were divided into a training group and a validation group at a 2:1 ratio using stochastic stratified sampling. Figure 1 shows the process of recruiting patients, including the inclusion and exclusion criteria and the number of patients. Clinical characteristics were gathered for each patient from medical records, and included age, menopausal status, and family history. Pathological data included estrogen receptor (ER), progesterone receptor (PR), and lymph node metastatic (LNM) status. The expression status of ER, PR, HER-2, and Ki-67 was determined with standard IHC (Gnant et al., 2011). The staining of cells indicates the expression status of pathological indicators. The ER and PR expressions were deemed positive if the number of ER or PR positive-stained nuclei was greater than 1%, and the expression level of Ki-67 was considered high if its positive staining rate was greater than 14% (Goldhirsch et al., 2011). Cases with IHC staining intensity confirmed as 3+ were defined as HER-2 positive, and cases with staining intensity of 2+ required fluorescence *in situ* hybridization (Fehrenbacher et al., 2020) analysis to determine whether they were HER-2 positive.

### 2.2 MRI scans and tumor segmentation

**FIGURE 2:**
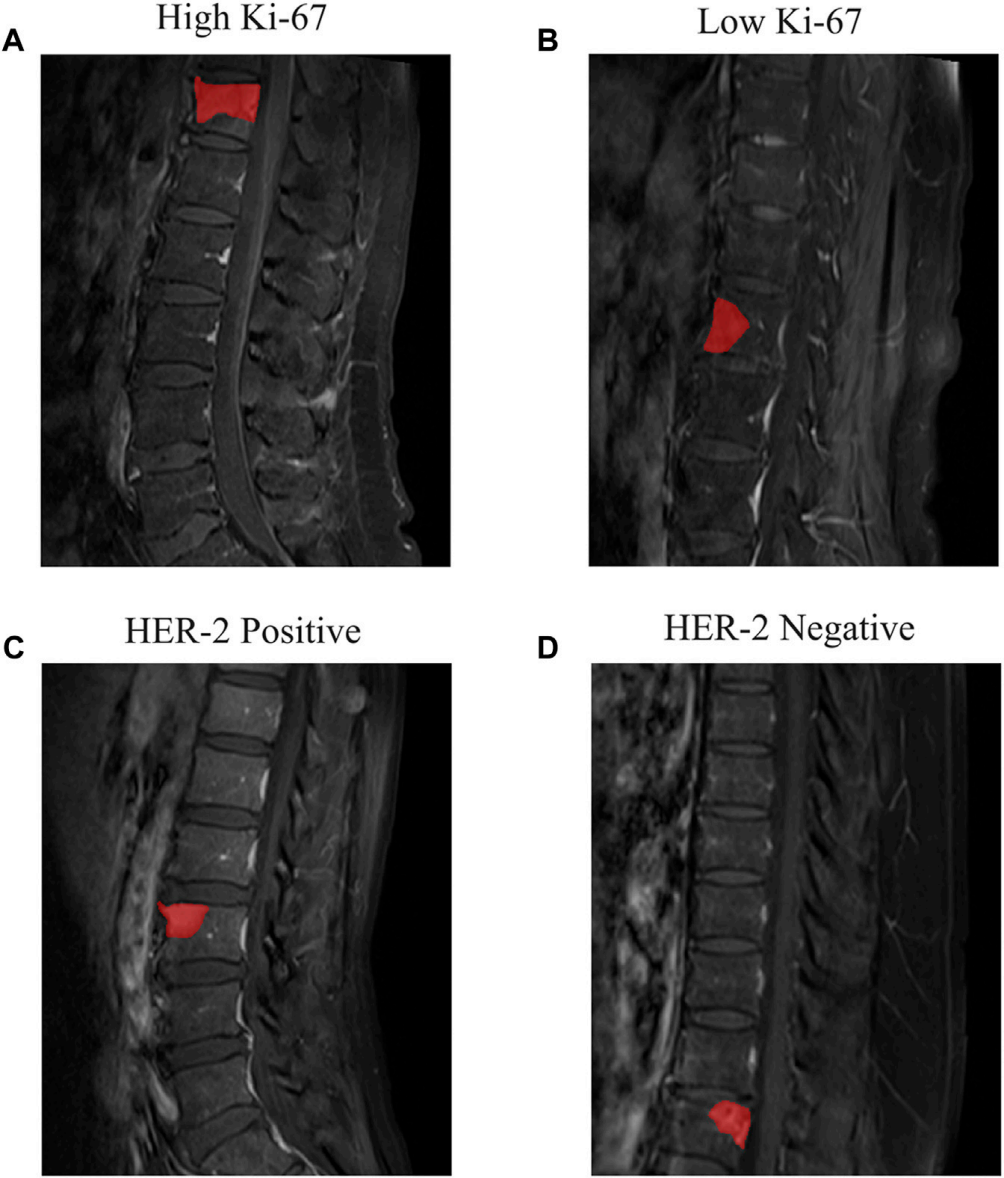
Examples of the T1CE MRI images of spine metastasis and segmented ROIs. **(A)** Patient with high Ki-67 expression level and **(B)** low Ki-67 expression level. **(C)** Patient with HER-2 positive and **(D)** HER-2 negative.

The sagittal T1CE-MRI data were obtained using a Siemens 3.0T MRI device (Verio, Siemens, Germany) with a repetition time of 420 m, echo time of 9 m, flip angle of 150°, acquisition matrix with dimensions of 320 × 272, field of view of 100 × 100 mm, and thickness of 4 mm. The T1CE MRI data were acquired by intravenous injection of Gadolinium-DTPA contrast agent (0.1 mmol/kg, Omniscan, GE Healthcare). The MRI data were stored in DICOM format on the picture archiving and communication system. The ITK-Snap software (v.3.8, available for download at www.itk-snap.org) was used by a radiologist with 4 years’ working experience to segment the region of interest (ROI) along the tumor border on the MRI images. The delineated ROIs were stored in NII (Data Format Working Group, 2004) format for further analysis. Figure 2 shows examples of manually delineated ROIs, including different levels of Ki-67 (Figures 2A, 2B) and different HER-2 status (Figures 2C, 2D).

### 2.3 Radiomics feature calculation

The radiomics features were all calculated using the PyRadiomics package (van Griethuysen et al., 2017). This is a comprehensive open-source platform that processes and extracts radiomics features from medical images using a large set of engineered hard-coded feature algorithms. The radiomics features are extracted in a four-step process: i) preprocessing of the images and ROIs; ii) application of enabled filters; iii) calculation of features; and iv) output of results. The two feature types are original features (first-order, shape and texture) and transformed features. The original features were calculated from the original MRI images, whereas the transformed features were calculated based on transformed MRI images obtained by applying various filters to the original images. In this study, the filter types used were the Exponential, Wavelet, Square, Squareroot, Local Binary Pattern, Logarithm, Gradient, and Laplacian of Gaussian filters. More information on the image feature extraction process can be found in the PyRadiomics documentation (https://pyradiomics.readthedocs.io/).

### 2.4 Identification of the most predictive features

To assess the reliability of the radiomics features and to exclude unstable features, 30 patients’ data were randomly selected for intraclass correlation coefficient (ICC) analysis (Koo and Li, 2016). The features with an intraclass correlation coefficient of greater than 0.80 were further selected by least absolute shrinkage and selection operator (LASSO) regression with 10-fold cross-validation. The training set was used to fit the LASSO regression model, and the sparsity of features was controlled by adjusting the regularization parameter lambda during the fitting process. The coefficients of all features were obtained from the trained LASSO regression model. A larger lambda value will result in more features having a coefficient of zero, thereby reducing the complexity of the model and the risk of overfitting (Sauerbrei et al., 2007). The value of lambda was computed at the position of one standard error from the maximum AUC (area under the receiver operating characteristic (ROC) curve), then the regression coefficient was determined and the valuable features were screened.

### 2.5 Development and validation of the radiomics signature

**FIGURE 3:**
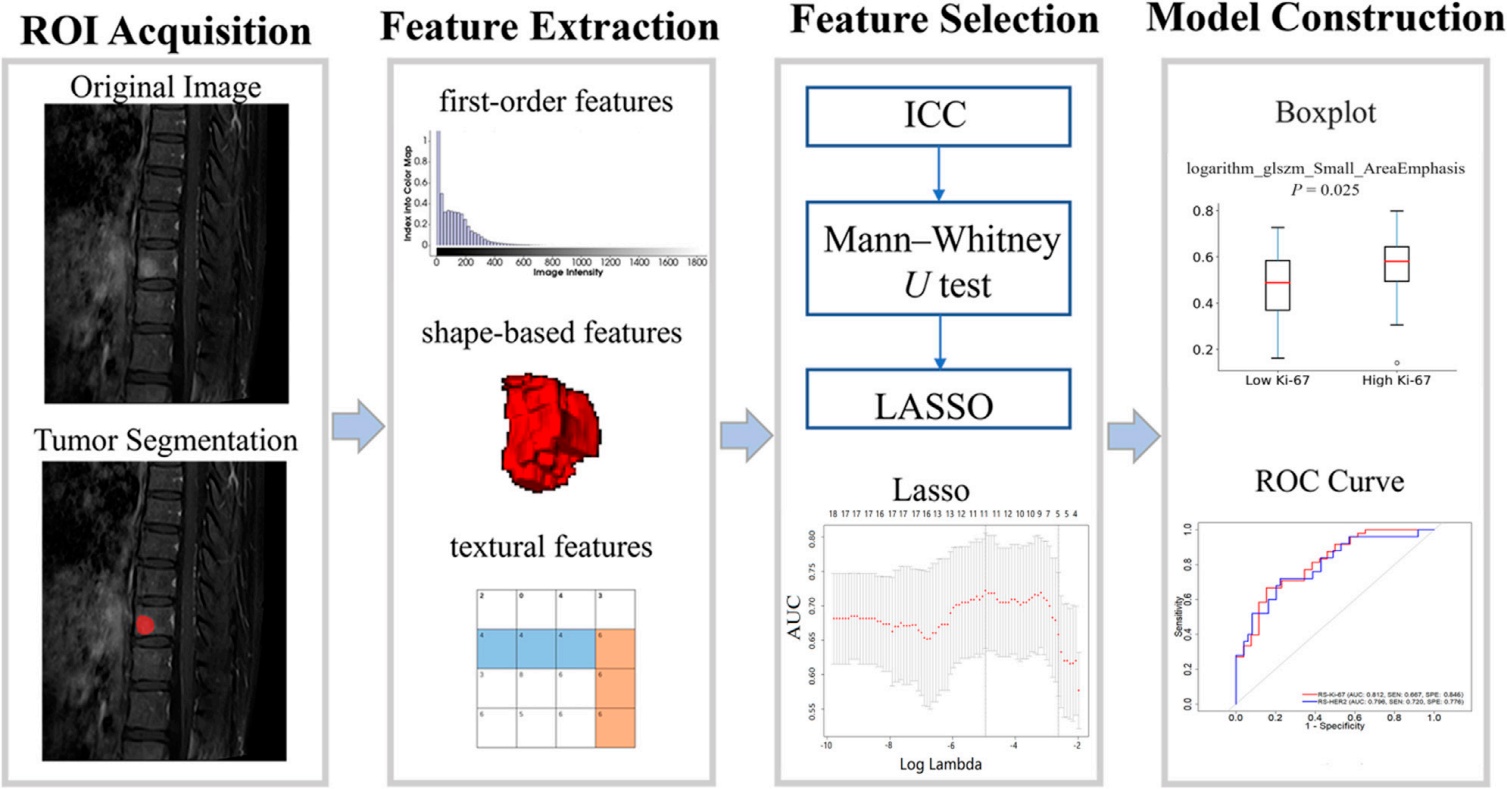
Overview of the study design.

The radiomics signature (RS) formula was calculated by integrating the final set of radiomics features and their corresponding coefficients using the *glmnet* package (Friedman et al., 2010) in R v.3.6. The performance of the RSs was assessed by ROC curve analysis, with the optimal cutoff values determined by the maximum Younden index (Ruopp et al., 2008) using the *sklearn* and *matplotlib* packages in Python v.3.6. The AUC values for the features were calculated based on logistic regression using the *pROC* package in R. Figure 3 depicts the workflow of this research, including ROI acquisition, feature extraction, feature selection, and model construction.

### 2.6 Statistical analysis

To identify statistically significant differences in the clinical and pathological characteristics between the high- and low-level Ki-67 and HER-2 positive/negative groups, the Chi-Square test and Fisher’s exact test were used to compare categorical variables. The normality of continuous variables was verified by the Shapiro–Wilk test. The Student’s t-test and Mann–Whitney U test were used to evaluate the continuous values. The hypothesis tests were two-sided with statistical significance set at 0.05.

## 3 Results

### 3.1 Patients’ characteristics

Table 1 presents statistical results regarding the patients’ characteristics. Between the high-level Ki-67 and low-level Ki-67 groups, the age was found to be significantly different (*p* < 0.05). Between the HER-2 positive and negative groups, no significant differences were observed (*p* > 0.05), although the age produced *p* < 0.05 in the training cohort.

**TABLE 1:** Characteristics of patients with spinal metastasis from primary BC.

Characteristic	Training cohort (*n* = 74)			Validation cohort (*n* = 36)			Training cohort (*n* = 74)			Validation cohort (*n* = 36)		
	High Ki-67 (*n* = 48)	Low Ki-67 (*n* = 26)	*P*	High Ki-67 (n = 18)	Low Ki-67 (*n* = 18)	*P*	HER-2 positive (*n* = 25)	HER-2 negative (*n* = 49)	*P*	HER-2 positive (*n* = 15)	HER-2 negative (*n* = 21)	*P*
Age (Mean ± SD)	54.48 ± 9.76	54.31 ± 9.06	^*^0.004	52.50 ± 9.22	49.06 ± 9.51	^*^0.006	49.73 ± 11.82	53.90 ± 8.70	^*^0.043	49.01 ± 9.23	56.57 ± 10.01	0.625
Menopausal status, No (%)			1.000			1.000			1.000			0.138
Premenopausal	7 (63.64)	4 (36.36)		3 (50.00)	3 (50.00)		4 (33.33)	8 (66.67)		4 (80.00)	1 (20.00)	
Postmenopausal	41 (65.08)	22 (34.92)		15 (50.00)	15 (50.00)		21 (33.87)	41 (66.13)		11 (35.48)	20 (64.52)	
Family history, No (%)			1.000			1.000			0.547			1.000
Yes	1 (50. 00)	1 (50.00)		0 (0.00)	1 (100.00)		0 (0.00)	2 (100.00)		0 (0.00)	1 (100.00)	
No	47 (65.28)	25 (34.72)		18 (51.43)	17 (48.57)		25 (34.72)	47 (65.28)		15 (42.86)	20 (57.14)	
LNM, No (%)			0.773			1.000			0.358			0.443
Yes	34 (62.96)	20 (37.04)		12 (50.00)	12 (50.00)		15 (29.41)	36 (70.59)		10 (37.04)	17 (62.96)	
No	14 (70.00)	6 (30.00)		6 (50.00)	6 (50.00)		10 (43.48)	13 (56.52)		5 (55.56)	4 (44.44)	
ER, No (%)			0.064			0.443			0.113			0.260
Positive	27 (56.25)	21 (43.75)		12 (44.44)	15 (55.56)		13 (26.53)	36 (73.47)		9 (34.62)	17 (65.38)	
Negative	21 (80.77)	5 (19.23)		6 (66.67)	3 (33.33)		12 (48.00)	13 (52.00)		6 (60.00)	4 (40.00)	
PR, No (%)			0.165			0.499			0.072			0.864
Positive	20 (55.56)	16 (44.44)		9 (42.86)	12 (57.14)		8 (22.22)	28 (77.78)		8 (38.10)	13 (61.90)	
Negative	28 (73.68)	10 (26.32)		9 (60.00)	6 (40.00)		17 (44.74)	21 (55.26)		7 (46.67)	8 (53.33)	
Histological grade, No (%)			0.166			0.309			0.609			0.957
Ⅰ	0 (0.00)	1 (100.00)		0 (0.00)	0 (0.00)		0 (0.00)	1 (100.00)		0 (0.00)	0 (0.00)	
Ⅱ	40 (63.49)	23 (36.51)		15 (46.87)	17 (53.13)		23 (35.94)	41 (64.06)		13 (41.94)	18 (58.06)	
Ⅲ	8 (80.00)	2 (20.00)		3 (75.00)	1 (25.00)		2 (22.22)	7 (77.78)		2 (40.00)	3 (60.00)	

HER-2, human epidermal growth factor receptor 2; Ki-67, antigen identified by monoclonal antibody; SD, standard deviation; LNM, lymph node metastasis; ER, estrogen receptor; PR, progesterone receptor.

*Statistically significant values of *p* < 0.05.

### 3.2 Radiomics feature selection

**FIGURE 4:**
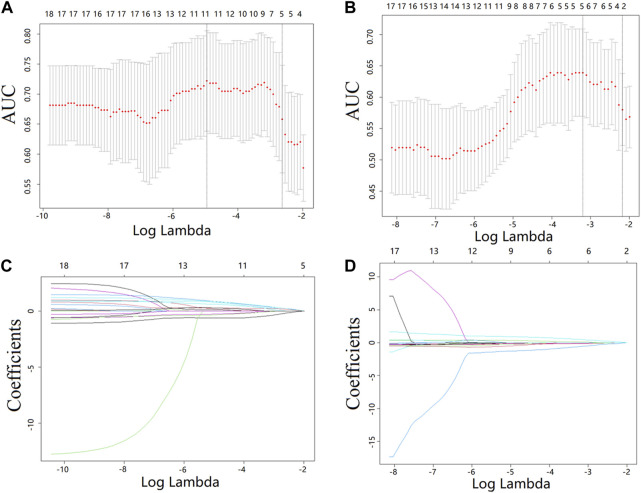
Feature selection from the T1CE MRI data with LASSO. **(A,B)** LASSO coefficient analysis of the features with 10-fold cross-validation to select optimal lambda for predicting the Ki-67 level **(A)** and HER-2 status **(B)**. **(C,D)** LASSO coefficients against the lambda, with five and two nonzero coefficients generated from the T1CE MRI data for predicting the Ki-67 level **(C)** and HER2 status **(D)**, respectively.

**FIGURE 5:**
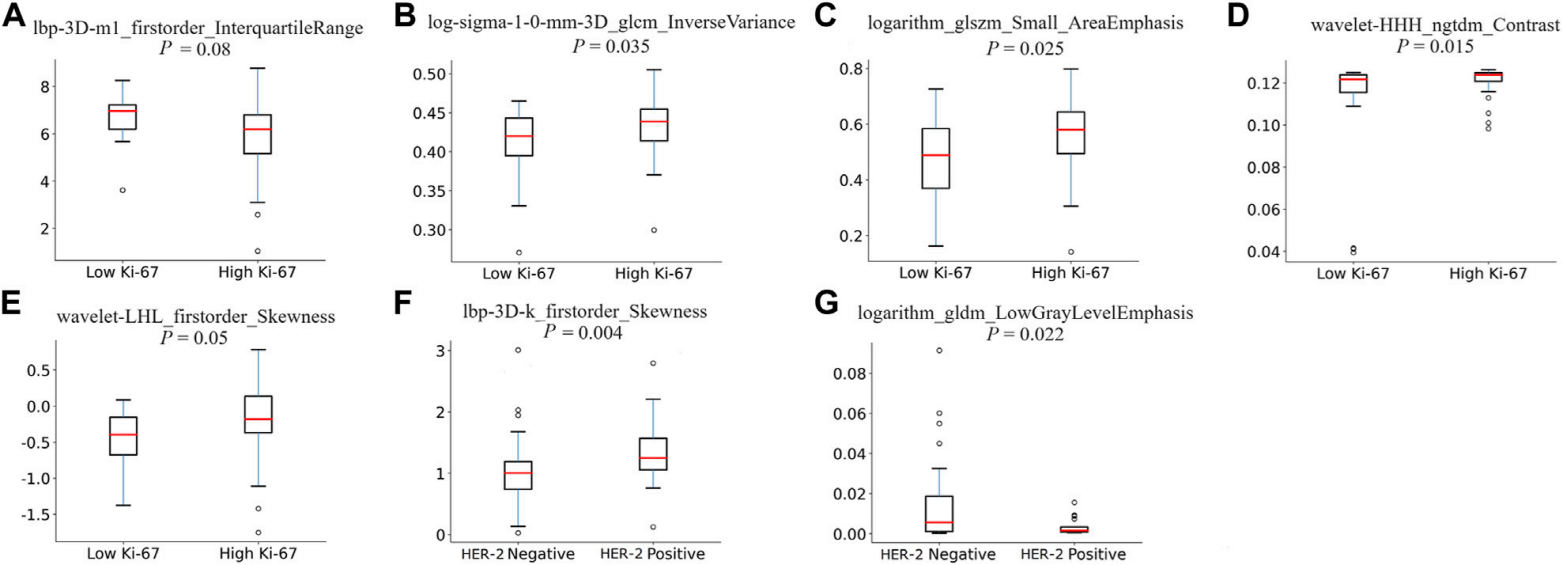
Boxplots of the selected features for predicting the Ki-67 level **(A–E)** and HER-2 status **(F, G)**.

The most predictive radiomics features were selected from the T1CE MRI of the spinal metastasis. Figure 4 depicts the feature selection process with LASSO (Sauerbrei et al., 2007). LASSO regression determines the most valuable features by selecting the appropriate regularization parameter lambda. To predict the Ki-67 level and HER-2 status, five and two features were finally selected, respectively. Table 2 lists the prediction performance of each of these features. Two features have *p*-values of less than 0.05 in both the training and validation cohorts. Figure 5 shows boxplots of the selected features, describing the maximum, minimum, median, and upper/lower quartiles, as well as the outliers. A detailed explanation of each selected feature is shown in Supplementary Table S1.

**TABLE 2:** Performance of the selected features for predicting the Ki-67 level and HER-2 status.

Biomarkers	Features	Cohorts	Mean ± SD		AUC	*P*
Ki-67	lbp-3D-m1_firstorder_InterquartileRange	Training	6.59 ± 1.12	5.85 ± 1.49	0.686	^*^0.008
		Validation	6.30 ± 1.83	7.02 ± 1.02	0.702	^*^0.038
	log-sigma-1-0-mm-3D_glcm_InverseVariance	Training	0.41 ± 0.04	0.43 ± 0.03	0.650	^*^0.035
		Validation	0.41 ± 0.05	0.42 ± 0.02	0.528	0.788
	logarithm_glszm_SmallAreaEmphasis	Training	0.48 ± 0.15	0.56 ± 0.12	0.659	^*^0.025
		Validation	0.58 ± 0.11	0.54 ± 0.12	0.580	0.420
	wavelet-HHH_ngtdm_Contrast	Training	0.11 ± 0.03	0.12 ± 0.01	0.672	^*^0.015
		Validation	0.12 ± 0.01	0.11 ± 0.03	0.565	0.617
	wavelet-LHL_firstorder_Skewness	Training	−0.46 ± 0.39	−0.18 ± 0.50	0.699	^*^0.005
		Validation	−0.62 ± 0.45	−0.24 ± 0.40	0.744	^*^0.013
HER-2	lbp-3D-k_firstorder_Skewness	Training	0.92 ± 0.42	1.18 ± 0.28	0.706	^*^0.004
		Validation	1.120 ± 0.45	0.97 ± 0.65	0.660	0.109
	logarithm_gldm_LowGrayLevelEmphasis	Training	0.01 ± 0.02	0.01 ± 0.01	0.664	^*^0.022
		Validation	0.01 ± 0.02	0.02 ± 0.02	0.667	0.095

SD, standard deviation; AUC, area under the ROC curve; Ki-67, antigen identified by monoclonal antibody; HER-2, human epidermal growth factor receptor 2.

*Statistically significant values of *p* < 0.05.

### 3.3 Development of the RSs

The finally selected MRI features were used to build the RSs for predicting the Ki-67 level (RS-Ki-67) and HER-2 (RS-HER-2) status. The RSs were established based on the selected radiomics features weighted by the respective LASSO coefficients. The formulas for the RSs are as follows:

RS-Ki-67 = 0.6505 - wavelet-HHH_ngtdm_Contrast × 0.2005 + wavelet-LHL_firstorder_Skewness × 0.1802 + lbp-3D-m1_firstorder_InterquartileRange × 0.1750 + log-sigma-1-0-mm-3D_glcm_InverseVariance × 0.2552 + logarithm_glszm_SmallAreaEmphasis × 0.2373.

RS-HER-2 =−0.6762 + logarithm_gldm_LowGrayLevelEmphasis × 0.0960–lbp-3D-k_firstorder_Skewness × 0.0866.

**FIGURE 6:**
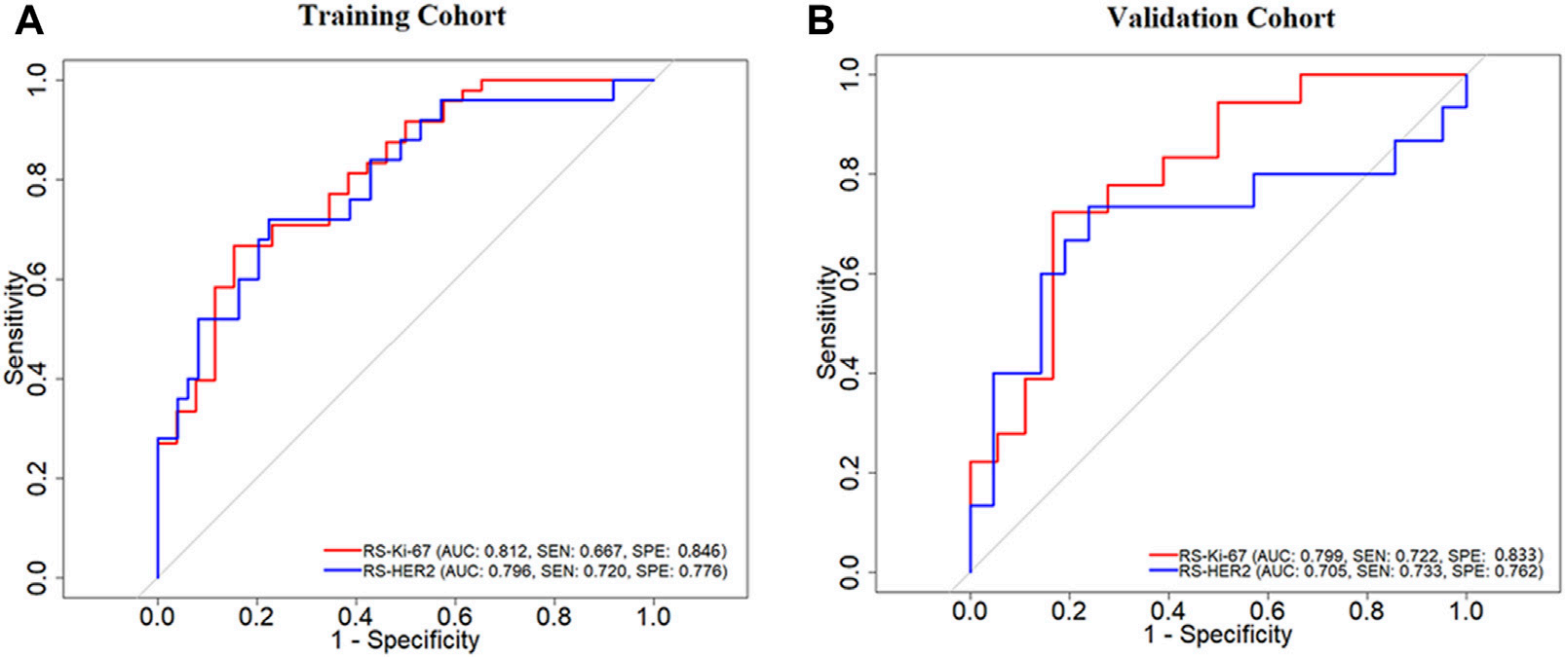
ROC curves of the developed RS-Ki-67 and RS-HER-2 for predicting the Ki-67 level and HER-2 status in the training **(A)** and validation **(B)** cohorts.

Figure 6 depicts the ROC curves of the developed RSs, where the horizontal axis represents the false positive rate and the vertical axis represents the true positive rate. The AUC was used to evaluate the classification performance of the models. As listed in Table 3, RS-Ki-67 generated good prediction performance, with AUCs of 0.812 (sensitivity (SEN) = 0.667 and specificity (SPE) = 0.846) in the training group and 0.799 (SEN = 0.722 and SPE = 0.833) in the validation group. RS-HER-2 also generated good prediction performance, with AUCs of 0.796 (SEN = 0.720 and SPE = 0.776) in the training group and 0.705 (SEN = 0.733 and SPE = 0.762) in the validation group.

**TABLE 3:** Performance of the developed RS-Ki-67 and RS-HER-2.

Model	Training cohort			Validation cohort		
	AUC (95% CI)	SEN	SPE	AUC (95% CI)	SEN	SPE
RS-Ki-67	0.812 (0.710–0.914)	0.667	0.846	0.799 (0.652–0.947)	0.722	0.833
RS-HER-2	0.796 (0.686–0.906)	0.720	0.776	0.705 (0.506–0.904)	0.733	0.762

AUC, area under the ROC curve; CI, confidence interval; SEN, sensitivity; SPE, specificity; Ki-67, antigen identified by monoclonal antibody; HER-2, human epidermal growth factor receptor 2.

## 4 Discussion

Early identification of molecular subtypes is essential for treatment in BC cases. Although there have been many studies on this topic, all have focused on the primary tumor (Ye et al., 2020). In clinical practice, however, we frequently receive BC patients carrying metastases whose primary tumor has already been surgically removed. Many of these patients lack complete records of molecular subtypes because the resection of the primary BC was previously performed in a county-level hospital. Noninvasive use of the metastasis to reflect the molecular subtype status provides an alternative, but this has not yet been investigated.

We found that both the Ki-67 level and HER-2 status can be assessed based on the spinal bone MRI. The developed RS-Ki-67 generated predictive AUCs of 0.812 and 0.799 on the training and validation cohorts, respectively; for RS-HER-2, the corresponding AUCs were 0.796 and 0.705. These values are lower than the results generated in recent MRI-based studies on primary BC (Fan et al., 2019; Li et al., 2021; Jiang et al., 2022b). We found that RS-Ki-67 always outperforms RS-HER-2 for predicting the Ki-67 level and HER-2 status in both the training and validation groups. This may be because Ki-67 expression reflects the cell proliferation ability, thus resulting in more obvious signal changes within the metastatic tumor. In contrast, HER-2 reflects the expression of receptors on the tumor cell surface and may produce smaller changes in the MRI signal. Our findings are partially in accordance with a recent comparison study on primary BC, which indicated that MRI-based radiomics are better at identifying high-level Ki-67 patients than HER-2 positive patients (Li et al., 2021).

From spinal MRI data, we calculated a total of 1967 radiomics features, and identified the five and two most important features for predicting the Ki-67 level and HER-2 status, respectively. Three of these seven features belong to the first-order feature category, and the other four belong to the texture feature category. The first-order features describe the distribution of signal intensity within the tumor, reflecting the heterogeneity of the tumor; texture features quantify the texture patterns and spatial distribution information inside tumors through texture matrices (Tagliafico et al., 2019; Jiang et al., 2022a). Our findings indicate that Ki-67 levels and HER-2 status are related to the heterogeneity and spatial complexity of tumors. These findings are partially in line with previous studies on primary tumors, which also indicated a strong relationship between the textural/first-order information and the Ki-67/HER-2 status in BC (Fan et al., 2017; Li et al., 2021; Niu et al., 2022). Additionally, our results may explain why visual inspection of spinal MRI images by radiologists struggles to determine the molecular subtype status, i.e., all predictive features are transformed features that are hidden in the high-dimensional space, and therefore cannot be recognized by humans.

Age was found to be related to the Ki-67 level. Although this result is not supported by several previous studies (Fan et al., 2019; Li et al., 2021; Niu et al., 2022), it is consistent with at least one prior conclusion (Son et al., 2020). We believe this is caused by the limited number of enrolled patients. Although the developed RSs produced acceptable AUCs, the predictive sensitivities were still low, especially for RS-HER-2, compared with a previous study on primary BC (Weigelt et al., 2005). The findings of this study are encouraging, and may widen the understanding of assessment for molecular subtypes and reveal the prediction efficiency of metastasis from primary BC.

There are several limitations to our study. The first issue is the small sample size, with all data obtained from a single center. The reliability of the identified features and RSs should be validated on multi-center data in future work. Second, we only analyzed the T1CE MRI on the bone metastasis. The T2-weighted fat-suppressed fast spin echo sequence should be further studied because this can suppress the fat hyperintensities of yellow bone marrow and may reflect the metastasis heterogeneity. Third, some other tumor markers (ER and PR) that are important for the prognosis and treatment of BC were not studied due to problems associated with data collection. Finally, the primary BC was not evaluated for comparison because of incomplete data, which should be addressed in future research.

## 5 Conclusion

This study has revealed that radiomics features derived from MRI images of bone metastasis from primary BC are predictive of the Ki-67 level and HER-2 status. The developed RSs, which integrate predictive MRI features, have the potential to be used as noninvasive tools for the assessment of molecular subtypes in BC.

## Data Availability

The original contributions presented in the study are included in the article/Supplementary Material, further inquiries can be directed to the corresponding authors.

## Abbreviations

AUC, area under the receiver operating characteristic curve; BC, breast cancer; CI, confidence interval; ER, estrogen receptor; HER-2, human epidermal growth factor receptor 2; ICC, intraclass correlation coefficient; IHC, immunohistochemistry; Ki-67, antigen identified by monoclonal antibody; LNM, lymph node metastatic; LASSO, least absolute shrinkage and selection operator; pCR, pathological complete response; PR, progesterone receptor; MRI, magnetic resonance imaging; ROI, region of interest; ROC, receiver operating characteristic; RS, radiomics signature; SD, standard deviation; SEN, sensitivity; SPE, specificity; T1CE, T1-weighted contrast-enhanced.

## References

[B1] Allemani C. Weir H. K. Carreira H. Harewood R. Spika D. Wang X. S. (2015). Global surveillance of cancer survival 1995-2009: analysis of individual data for 25,676,887 patients from 279 population-based registries in 67 countries (CONCORD-2). Lancet 385 (9972), 977–1010. 10.1016/S0140-6736(14)62038-9 25467588 PMC4588097

[B2] Cheang M. C. Chia S. K. Voduc D. Gao D. Leung S. Snider J. (2009). Ki67 index, HER2 status, and prognosis of patients with luminal B breast cancer. J. Natl. Cancer Inst. 101 (10), 736–750. 10.1093/jnci/djp082 19436038 PMC2684553

[B3] Data Format Working Group (2004). NIfTI: neuroimaging informatics technology initiative . https://nifti.nimh.nih.gov/(Accessed December 5, 2023).

[B4] Fan M. Li H. Wang S. Zheng B. Zhang J. Li L. (2017). Radiomic analysis reveals DCE-MRI features for prediction of molecular subtypes of breast cancer. PLoS One 12 (2), e0171683. 10.1371/journal.pone.0171683 28166261 PMC5293281

[B5] Fan M. Zhang P. Wang Y. Peng W. Wang S. Gao X. (2019). Radiomic analysis of imaging heterogeneity in tumours and the surrounding parenchyma based on unsupervised decomposition of DCE-MRI for predicting molecular subtypes of breast cancer. Eur. Radiol. 29 (8), 4456–4467. 10.1007/s00330-018-5891-3 30617495

[B6] Fehrenbacher L. Cecchini R. S. Geyer C. E. Jr. Rastogi P. Costantino J. P. Atkins J. N. (2020). NSABP B-47/NRG oncology phase III randomized trial comparing adjuvant chemotherapy with or without trastuzumab in high-risk invasive breast cancer negative for HER2 by FISH and with IHC 1+ or 2. J. Clin. Oncol. 38 (5), 444–453. 10.1200/JCO.19.01455 31821109 PMC7007289

[B7] Foerster R. Bruckner T. Bostel T. Schlampp I. Debus J. Rief H. (2015). Prognostic factors for survival of women with unstable spinal bone metastases from breast cancer. Radiat. Oncol. 10, 144. 10.1186/s13014-015-0458-9 26169373 PMC4501125

[B8] Friedman J. Hastie T. Tibshirani R. (2010). Regularization paths for generalized linear models via coordinate descent. J. Stat. Softw. 33 (1), 1–22. 10.18637/jss.v033.i01 20808728 PMC2929880

[B9] Gillies R. J. Kinahan P. E. Hricak H. (2016). Radiomics: images are more than pictures, they are data. Radiology 278 (2), 563–577. 10.1148/radiol.2015151169 26579733 PMC4734157

[B10] Gnant M. Harbeck N. Thomssen C. (2011). St. Gallen 2011: summary of the consensus discussion. Breast Care (Basel) 6 (2), 136–141. 10.1159/000328054 21633630 PMC3100376

[B11] Goldhirsch A. Wood W. C. Coates A. S. Gelber R. D. Thurlimann B. Senn H. J. (2011). Strategies for subtypes--dealing with the diversity of breast cancer: highlights of the st. Gallen international expert consensus on the primary therapy of early breast cancer 2011. Ann. Oncol. 22 (8), 1736–1747. 10.1093/annonc/mdr304 21709140 PMC3144634

[B12] Hagberg K. W. Taylor A. Hernandez R. K. Jick S. (2013). Incidence of bone metastases in breast cancer patients in the United Kingdom: results of a multi-database linkage study using the general practice research database. Cancer Epidemiol. 37 (3), 240–246. 10.1016/j.canep.2013.01.006 23416031

[B13] Hosny A. Parmar C. Quackenbush J. Schwartz L. H. Aerts H. (2018). Artificial intelligence in radiology. Nat. Rev. Cancer 18 (8), 500–510. 10.1038/s41568-018-0016-5 29777175 PMC6268174

[B14] Janjan N. Lutz S. T. Bedwinek J. M. Hartsell W. F. Ng A. Pieters R. S. Jr. (2009). Therapeutic guidelines for the treatment of bone metastasis: a report from the American college of radiology appropriateness criteria expert panel on radiation oncology. J. Palliat. Med. 12 (5), 417–426. 10.1089/jpm.2009.9633 19416037

[B15] Jiang T. Jiang W. Chang S. Wang H. Niu S. Yue Z. (2022a). Intratumoral analysis of digital breast tomosynthesis for predicting the Ki-67 level in breast cancer: a multi-center radiomics study. Med. Phys. 49 (1), 219–230. 10.1002/mp.15392 34861045

[B16] Jiang T. Song J. Wang X. Niu S. Zhao N. Dong Y. (2022b). Intratumoral and peritumoral analysis of mammography, tomosynthesis, and multiparametric MRI for predicting ki-67 level in breast cancer: a radiomics-based study. Mol. Imaging Biol. 24 (4), 550–559. 10.1007/s11307-021-01695-w 34904187

[B17] Kim K. I. Lee K. H. Kim T. R. Chun Y. S. Lee T. H. Park H. K. (2014). Ki-67 as a predictor of response to neoadjuvant chemotherapy in breast cancer patients. J. Breast Cancer 17 (1), 40–46. 10.4048/jbc.2014.17.1.40 24744796 PMC3988341

[B18] Koo T. K. Li M. Y. (2016). A guideline of selecting and reporting intraclass correlation coefficients for reliability research. J. Chiropr. Med. 15 (2), 155–163. 10.1016/j.jcm.2016.02.012 27330520 PMC4913118

[B19] Lambin P. Leijenaar R. T. H. Deist T. M. Peerlings J. de Jong E. E. C. van Timmeren J. (2017). Radiomics: the bridge between medical imaging and personalized medicine. Nat. Rev. Clin. Oncol. 14 (12), 749–762. 10.1038/nrclinonc.2017.141 28975929

[B20] Leithner D. Mayerhoefer M. E. Martinez D. F. Jochelson M. S. Morris E. A. Thakur S. B. (2020). Non-invasive assessment of breast cancer molecular subtypes with multiparametric magnetic resonance imaging radiomics. J. Clin. Med. 9 (6), 1853. 10.3390/jcm9061853 32545851 PMC7356091

[B21] Li C. Song L. Yin J. (2021). Intratumoral and peritumoral radiomics based on functional parametric maps from breast DCE-MRI for prediction of HER-2 and ki-67 status. J. Magn. Reson Imaging 54 (3), 703–714. 10.1002/jmri.27651 33955619

[B22] Loibl S. Gianni L. (2017). HER2-positive breast cancer. Lancet 389 (10087), 2415–2429. 10.1016/S0140-6736(16)32417-5 27939064

[B23] Loibl S. Poortmans P. Morrow M. Denkert C. Curigliano G. (2021). Breast cancer. Lancet 397 (10286), 1750–1769. 10.1016/S0140-6736(20)32381-3 33812473

[B24] Niu S. Jiang W. Zhao N. Jiang T. Dong Y. Luo Y. (2022). Intra- and peritumoral radiomics on assessment of breast cancer molecular subtypes based on mammography and MRI. J. Cancer Res. Clin. Oncol. 148 (1), 97–106. 10.1007/s00432-021-03822-0 34623517 PMC11800953

[B25] Ruopp M. D. Perkins N. J. Whitcomb B. W. Schisterman E. F. (2008). Youden Index and optimal cut-point estimated from observations affected by a lower limit of detection. Biom J. 50 (3), 419–430. 10.1002/bimj.200710415 18435502 PMC2515362

[B26] Sauerbrei W. Royston P. Binder H. (2007). Selection of important variables and determination of functional form for continuous predictors in multivariable model building. Stat. Med. 26 (30), 5512–5528. 10.1002/sim.3148 18058845

[B27] Son J. Lee S. E. Kim E. K. Kim S. (2020). Prediction of breast cancer molecular subtypes using radiomics signatures of synthetic mammography from digital breast tomosynthesis. Sci. Rep. 10 (1), 21566. 10.1038/s41598-020-78681-9 33299040 PMC7726048

[B28] Sutton E. J. Dashevsky B. Z. Oh J. H. Veeraraghavan H. Apte A. P. Thakur S. B. (2016). Breast cancer molecular subtype classifier that incorporates MRI features. J. Magn. Reson Imaging 44 (1), 122–129. 10.1002/jmri.25119 26756416 PMC5532744

[B29] Tagliafico A. S. Bignotti B. Rossi F. Matos J. Calabrese M. Valdora F. (2019). Breast cancer Ki-67 expression prediction by digital breast tomosynthesis radiomics features. Eur. Radiol. Exp. 3 (1), 36. 10.1186/s41747-019-0117-2 31414273 PMC6694353

[B30] van Griethuysen J. J. M. Fedorov A. Parmar C. Hosny A. Aucoin N. Narayan V. (2017). Computational radiomics system to decode the radiographic phenotype. Cancer Res. 77 (21), e104–e107. 10.1158/0008-5472.CAN-17-0339 29092951 PMC5672828

[B31] van Ramshorst M. S. Loo C. E. Groen E. J. Winter-Warnars G. H. Wesseling J. van Duijnhoven F. (2017). MRI predicts pathologic complete response in HER2-positive breast cancer after neoadjuvant chemotherapy. Breast Cancer Res. Treat. 164 (1), 99–106. 10.1007/s10549-017-4254-0 28432515

[B32] Weigelt B. Peterse J. L. van 't Veer L. J. (2005). Breast cancer metastasis: markers and models. Nat. Rev. Cancer 5 (8), 591–602. 10.1038/nrc1670 16056258

[B33] Wiesner F. G. Magener A. Fasching P. A. Wesse J. Bani M. R. Rauh C. (2009). Ki-67 as a prognostic molecular marker in routine clinical use in breast cancer patients. Breast 18 (2), 135–141. 10.1016/j.breast.2009.02.009 19342238

[B34] Ye D. M. Wang H. T. Yu T. (2020). The application of radiomics in breast MRI: a review. Technol. Cancer Res. Treat. 19, 1533033820916191. 10.1177/1533033820916191 32347167 PMC7225803

[B35] Yerushalmi R. Woods R. Ravdin P. M. Hayes M. M. Gelmon K. A. (2010). Ki67 in breast cancer: prognostic and predictive potential. Lancet Oncol. 11 (2), 174–183. 10.1016/S1470-2045(09)70262-1 20152769

