# Supplementary material for: Radiomics signatures for predicting the Ki-67 level and HER-2 status based on bone metastasis from primary breast cancer
Source: Front Cell Dev Biol. 2024 Jan 8;11:1220320. doi: 10.3389/fcell.2023.1220320 (PMC10804450; doi:10.3389/fcell.2023.1220320)
Supplement: Supplementary file 1 [file Table1.DOCX]

Supplementary Material

**Supplementary Table S1**

**Table S1**. Detailed explanations of the selected features.

| **Feature Name** | **Feature Type** | **Explanation** |
| --- | --- | --- |
| lbp-3D-m1_firstorder_InterquartileRange | first-order feature | The interquartile range of the first-order statistical histogram within the ROI. |
| log-sigma-1-0-mm-3D_glcm_InverseVariance | texture feature | The inverse variance of Gray Level Co-occurrence Matrix. |
| logarithm_glszm_SmallAreaEmphasis | texture feature | Small Area Emphasis of GLSZM is a measure of the distribution of size zones, with a greater value indicative of more smaller size zones and more fine textures. |
| wavelet-HHH_ngtdm_Contrast | texture feature | Contrast of NGTDM is a measure of the spatial intensity change, depending on the overall gray level dynamic range. Contrast is high when both the dynamic range and the spatial change rate are high. |
| wavelet-LHL_firstorder_Skewness | first-order feature | Skewness of the first-order statistical histogram measures the asymmetry of the distribution of values about the Mean value. Depending on where the tail is elongated and the mass of the distribution is concentrated, this value can be positive or negative. |
| lbp-3D-k_firstorder_Skewness | first-order feature | Skewness of the first-order statistical histogram measures the asymmetry of the distribution of values about the Mean value. |
| logarithm_gldm_LowGrayLevelEmphasis | texture feature | Low Gray Level Emphasis of GLDM measures the distribution of low gray-level values, with a higher value indicating a greater concentration of low gray-level values in the image. |
